# Emerging Bio-Intelligent Dental Prostheses: A Shift Toward Interactive and Adaptive Prosthodontic Systems

**DOI:** 10.7759/cureus.93483

**Published:** 2025-09-29

**Authors:** Akshim Rana, Shubham K Srivastava, Chinmoy Sikdar, Indrakumar HS, Aditya Shewale

**Affiliations:** 1 Department of Prosthodontics, Sardar Patel Post Graduate Institute of Dental and Medical Sciences, Lucknow, IND; 2 Department of Prosthodontics, College of Dental Science and Hospital, Indore, IND; 3 Department of Prosthodontics, SMBT Dental College and Hospital, Sangamner, IND

**Keywords:** bio-intelligent prostheses, digital dentistry, oral rehabilitation, prosthodontics, quality of life

## Abstract

Emerging bio-intelligent dental prostheses mark a paradigm shift from passive tissue replacements to interactive, adaptive systems. Traditional dentures and fixed prostheses restore function and esthetics but remain static within a dynamic oral environment. Recent advances in materials science, sensor technology, nanotechnology, and artificial intelligence (AI) are redefining prosthodontics by enabling prostheses to monitor, respond, and integrate with biological processes. Biosensor-embedded implants and smart dentures can track peri-implant tissue health, detect oral dryness, or provide real-time temperature data, while AI systems interpret inputs for predictive and preventive care. These innovations expand prosthodontics into a partner discipline of systemic healthcare. However, challenges remain, including device durability, power supply, data security, regulatory frameworks, and equitable access. This editorial explores the emerging concept of bio-intelligent prostheses, highlighting their potential applications, limitations, and implications for future prosthodontic education, practice, and interdisciplinary collaboration. Such systems position prostheses not as inert replacements but as intelligent health interfaces shaping the future of dental medicine.

## Editorial

Prosthodontics has long been defined by its role in replacing missing oral tissues with artificial substitutes designed to restore esthetics, function, and patient comfort. For decades, conventional dentures and fixed prostheses have served this purpose effectively, yet they have remained fundamentally passive, unable to respond to the constantly changing oral environment. The oral cavity, however, is far from static; it represents a highly dynamic ecosystem characterized by fluctuating occlusal forces, microbial challenges, salivary biochemical variations, and continuous remodeling of hard and soft tissues. While advances in digital dentistry, computer-aided design (CAD)/computer-aided manufacturing (CAM) workflows, and implantology have refined precision, efficiency, and treatment options, the underlying philosophy of prosthetic design has largely remained static. This incongruence between biological dynamism and prosthetic passivity has created a pressing need for a new paradigm, giving rise to the concept of bio-intelligent prostheses, devices that integrate sensing, processing, and adaptive responses to align with the oral and systemic environment [1-2].

The evolution of prosthodontics over the past century illustrates the trajectory from purely mechanical replacements to digitally enhanced, high-precision prostheses. Early removable appliances, from ivory dentures to vulcanite-based prostheses, were designed solely for mechanical function and esthetic restoration, with little consideration of biological interaction. The 20th century brought acrylic resin, casting technologies, and, later, osseointegrated implants, which transformed both the longevity and functional predictability of prosthetic treatment. The digital revolution of the late 20th and early 21st centuries, marked by CAD/CAM milling, 3D printing, and guided implant surgery, further enhanced the reproducibility and customization of prosthetic appliances [3]. Despite these advances, however, the core philosophy of prosthodontic rehabilitation remained largely unidimensional: the replacement of missing tissues without active engagement with the patient’s physiological or systemic state. This historical perspective underscores the essential gap that bio-intelligent prostheses aim to address, positioning the prosthesis not merely as a static replacement but as an interactive, adaptive interface between the human body and restorative technology [3].

The rationale for developing bio-intelligent prostheses emerges from the inherent complexity of the oral cavity as a biomechanical and biological system. Salivary composition, temperature, microbial balance, and occlusal forces fluctuate continuously, influenced by dietary intake, systemic health conditions, and aging [4]. Conventional prostheses, no matter how precise or well-fitted, are unable to sense or respond to these dynamic changes. Consequently, patients may experience discomfort, reduced prosthesis longevity, and an elevated risk of complications such as mucosal irritation, peri-implant inflammation, or occlusal imbalance. Bio-intelligent prostheses seek to bridge this gap by incorporating sensors capable of detecting mechanical, chemical, or thermal cues, combined with computational systems that analyze these inputs and initiate adaptive responses. The ultimate objective is to transform prostheses from passive restorations into active healthcare devices, capable of participating in both oral and systemic health monitoring [1-2,4].

Advances in materials science, biomedical engineering, and sensor miniaturization have laid the groundwork for bio-intelligent prosthodontics. A landmark contribution by Li et al. introduced the concept of embedding biosensor platforms within dental implant fixtures and abutments, enabling continuous peri-implant tissue monitoring with wireless data transmission [1]. This breakthrough shifted implants from being passive mechanical supports to active diagnostic devices, capable of signaling pathological changes well before clinical symptoms appear. Expanding on this idea, Nam et al. designed complete dentures equipped with thin-film temperature sensors fabricated via femtosecond laser pulses, allowing real-time assessment of intraoral thermal fluctuations relevant to both local oral health and systemic conditions [2]. Collectively, these technological milestones illustrate how prostheses are evolving from static mechanical replacements into dynamic, sensor-enabled biomedical systems.

The clinical potential of bio-intelligent prostheses is extensive, moving beyond restoration alone to integrated health surveillance. Dentures embedded with biosensors could track oral dryness, mucosal inflammation, or microbial shifts [5], while implant-supported prostheses might provide real-time insights into occlusal load distribution, peri-implant disease risk, and inflammatory biomarkers [1-2]. Such functionality bridges prosthetic rehabilitation with personalized health monitoring, creating opportunities for early intervention and improved patient care [4]. From a broader perspective, continuous non-invasive monitoring may strengthen preventive dentistry, minimize complication rates, and align prosthodontic practice with precision medicine [4]. Looking forward, these innovations position prostheses not merely as tools for functional replacement, but as proactive health instruments with systemic diagnostic value.

Nanotechnology further expands the potential of bio-intelligent prostheses by enabling the development of multifunctional surfaces and responsive biomaterials. Nanomaterial-based biosensors have been explored for detecting specific salivary biomarkers, releasing antimicrobial agents, and creating responsive implant coatings that can adapt to environmental changes [4]. These innovations blur the traditional boundaries between prosthodontics and systemic diagnostics, suggesting a future in which prostheses function not only as oral restoratives but also as personalized medical devices capable of contributing to early detection and monitoring of systemic conditions such as diabetes, cardiovascular disease, and cancer [4-5]. The integration of nanotechnology into prosthetic design offers additional advantages, including improved surface bioactivity, enhanced tissue integration, and the potential for controlled therapeutic release, reinforcing the multifunctional capabilities of future prosthetic systems [4].

Artificial intelligence (AI) serves as the computational backbone that enables prostheses to interpret and respond to complex biosensor data. Systematic reviews have demonstrated the utility of AI in prosthodontics for diagnosis, predictive modeling, and treatment planning [6-8]. When integrated into bio-intelligent prostheses, AI can analyze continuous streams of intraoral and systemic data to predict prosthesis wear, detect early pathological changes, monitor occlusal balance, and guide patient-specific interventions [7-8]. Such predictive capabilities align with the broader movement toward precision and preventive medicine, where interventions are tailored to individual biological responses rather than standardized protocols [6]. By coupling real-time biosensor data with intelligent computational analysis, prostheses could potentially alert patients and clinicians to impending complications, enhancing both patient engagement and clinical decision-making [6-7].

Despite the transformative potential of bio-intelligent prostheses, several technical and practical challenges must be addressed before widespread clinical adoption can occur. The long-term durability and biocompatibility of embedded electronics in the oral cavity remain uncertain, particularly in the presence of fluctuating pH, temperature, and masticatory forces [9-10]. Power supply constraints pose additional limitations, necessitating solutions such as wireless charging, energy harvesting from mechanical or thermal sources, or ultra-low-power sensor systems [1,4,10]. Data management and cybersecurity are equally critical, as continuous monitoring generates sensitive health information that must be protected in accordance with ethical, legal, and regulatory standards [4,6-7]. Failure to ensure secure handling of patient data could undermine both patient trust and clinical efficacy [6]. Furthermore, economic and social factors must be considered, as the cost and complexity of bio-intelligent prostheses may exacerbate disparities in access to care if affordability and scalability are not addressed [3].

The ethical and regulatory landscape for bio-intelligent prostheses adds another layer of complexity. These devices occupy the intersection between dental appliances and medical devices, raising questions regarding data ownership, informed consent, clinical responsibility, and regulatory oversight. Current frameworks for medical device approval, including Food and Drug Administration (FDA) and Conformité Européenne (CE) regulations, may require adaptation to accommodate multifunctional prosthetic systems that combine restorative, diagnostic, and potentially therapeutic functions [10]. In addition, clinicians and patients must navigate considerations related to privacy, AI interpretability, and ethical use of continuous monitoring technologies, emphasizing the need for guidelines and best practices tailored to this emerging domain [6-7].

From an educational perspective, the rise of bio-intelligent prostheses necessitates significant reforms in prosthodontic training. Clinicians must acquire foundational knowledge in AI, digital health, biosensor technology, and ethical data management [7]. Interdisciplinary collaboration with engineers, data scientists, and bioethicists will be essential to ensure responsible innovation and effective clinical translation [8]. Preparing the next generation of prosthodontists to critically evaluate and implement bio-intelligent systems will be a vital step toward realizing their full potential in both oral and systemic healthcare contexts.

Looking toward the future, the trajectory of bio-intelligent prosthetics suggests a gradual but inevitable transformation of prosthodontics. While fully closed-loop systems incorporating neural interfaces remain aspirational, the convergence of biosensors, nanotechnology, and AI already heralds a paradigm in which prostheses function as intelligent health interfaces. Such devices could monitor patient health in real-time, anticipate complications, and adapt to changing physiological conditions, effectively transforming prosthodontics from a primarily restorative discipline into a proactive, preventive, and integrative field within healthcare [3-5,7]. The collaboration between clinicians, engineers, researchers, and policymakers will be pivotal in shaping this future, ensuring that innovations are both clinically effective and ethically sound [4-5].

**Figure 1:**
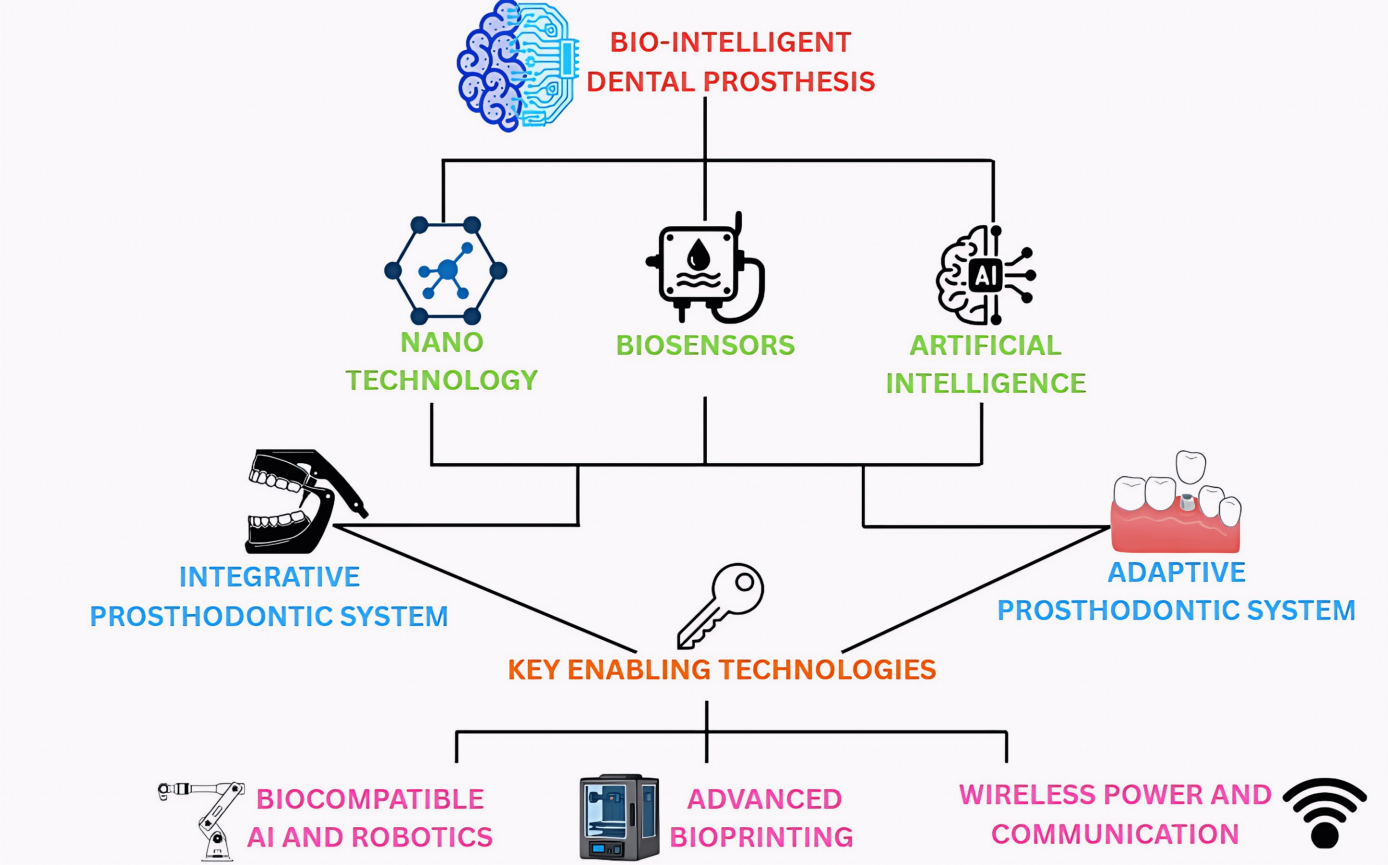
A conceptual framework for bio-intelligent dental prostheses This diagram illustrates the convergence of core disciplines like nanotechnology, biosensors, and artificial intelligence in the development of advanced dental prostheses. These fields contribute to the creation of both integrative and adaptive prosthodontic systems, which are underpinned by key enabling technologies such as biocompatible AI with robotics, advanced bioprinting, and wireless power and communication.

In conclusion, bio-intelligent prostheses represent a profound redefinition of the field of prosthodontics (Figure 1). Moving beyond passive restoration, these devices promise to actively participate in monitoring, maintaining, and potentially improving both oral and systemic health. Their integration of sensing technologies, adaptive materials, and intelligent computational analysis situates prosthodontics at the forefront of personalized, preventive healthcare. While technical, ethical, and economic challenges remain, the potential benefits of bio-intelligent prostheses are substantial, positioning the prosthesis not merely as a mechanical replacement but as a dynamic, interactive partner in patient health. By embracing these advances, prosthodontics can evolve to meet the demands of a changing healthcare landscape, ultimately reshaping the future of dental medicine.

Despite their promise, bio-intelligent dental prostheses remain largely in the conceptual or experimental stage, with several limitations impeding immediate translation into routine practice. Current evidence is predominantly derived from in vitro studies, pilot trials, and engineering prototypes, highlighting a lack of long-term clinical validation. Device miniaturization, durability in the harsh oral environment, and sustainable power supply systems remain unresolved engineering challenges. Furthermore, data security, ethical governance, and regulatory pathways for multifunctional prosthetic systems are underdeveloped, creating uncertainty around approval and clinical accountability. Accessibility and affordability also pose significant barriers, as advanced technologies risk widening disparities in oral healthcare delivery if not designed for scalability. Importantly, most existing studies emphasize feasibility rather than patient-centered outcomes, leaving gaps in understanding the real-world impact of bio-intelligent prostheses on quality of life, systemic health monitoring, and interdisciplinary care. Addressing these gaps through robust translational research, standardized clinical protocols, and collaborative regulatory frameworks will be crucial for realizing the full potential of this emerging paradigm.
